# Rubella Serosurvey Among Future Healthcare Workers

**DOI:** 10.3389/fpubh.2021.741178

**Published:** 2021-09-13

**Authors:** Andrea Trevisan, Paola Mason, Annamaria Nicolli, Stefano Maso, Chiara Bertoncello

**Affiliations:** Department of Cardiac Thoracic Vascular Sciences and Public Health, University of Padova (Italy), Padova, Italy

**Keywords:** rubella, rubella vaccine, rubella antibodies, medical students, healthcare workers

## Abstract

**Objective:** Rubella is a very diffusive but relatively benign infectious disease unless contracted during pregnancy, when it causes congenital rubella syndrome. The aim of this research was to determine the prevalence and titer of antirubella antibodies in a population of future healthcare workers (students at the school of medicine).

**Methods:** The cohort consisted of 11,022 students who underwent antibody analysis after the presentation of a vaccine certificate.

**Results:** Vaccination compliance was very high, particularly in younger students (born after 1995), reaching almost 100% (at least one dose). Unvaccinated students born before 1990 had high seropositivity (>95%), but this percentage dropped to zero among the youngest students. Variables affecting antibody titer included year of birth and sex. Considering only vaccinated students, a greater antibody response was observed if the vaccine was administered between 8 and 10 years of age. Female sex was associated with more significant (*p* < 0.0001) positivity and higher antibody titer after one and two doses. However, this difference appeared less consistent in relation to year of birth.

**Conclusions:** The studied population exhibited excellent vaccination compliance, high seropositivity, and high antibody titer. Vaccine and immune coverage were higher than what is deemed necessary to achieve herd immunity.

## Introduction

Rubella is an acute viral infection caused by an RNA togavirus (genus *Rubivirus*). A high percentage of rubella infections in both children and adults are subclinical, but rubella during pregnancy is associated with potentially serious complications for the fetus due to congenital rubella syndrome (CRS).

Since 1999, the Italian Board of Health has encouraged the measles, mumps, and rubella (MMR) vaccine, and a mass vaccination campaign was launched (1), even though the single rubella vaccine has been available since 1972. The rubella vaccine was actively offered to adolescent women during primary or secondary school from 1972 to the cohort 1988–1989. Notwithstanding the high percentage of immunity (acquired *via* disease or vaccination), relatively low compliance with vaccination is why rubella has continued to circulate in Italy and CRS still occurs (2). Fortunately, however, between 2005 and February 2018, only 88 cases of CRS were registered in Italy, and only 173 cases of rubella disease during pregnancy were reported (3).

In 2017, Italy approved the National Plan for Eradication of Measles and Congenital Rubella (4), according to the objectives of World Health Organization (WHO) 2012–2020 (5). Furthermore, according to the law established in 2017, the rubella vaccine (as MMR) is mandatory in Italy (6).

Moreover, the “National Vaccination Prevention Plan” 2017–2019 (4) recommends that healthcare workers (HCWs) be vaccinated against seven transmissible diseases, including rubella. Rubella immunity induced by vaccination appears to be persistent; therefore, routine booster immunizations do not seem to be necessary (7). However, a second immunization program should be considered to achieve high antibody-positivity rates and protect against primary vaccination failure. Several reports suggest that one dose can produce lifelong immunity (8) and that the vaccine induces a long-lasting antibody response of up to 21 years (9). The vaccination program carried out in Finland also eliminated CRS from that country (10).

The aim of this research was to evaluate the compliance with rubella vaccination, the percentage of seropositivity, and antibody titer according to the vaccination schedules (one or two doses) in a cohort of future HCWs recruited from a population of medical school students.

## Methods

### Population

A cohort of 11,022 students enrolled at the School of Medicine of the University of Padua (Italy) were recruited (2004–2020) according to the following inclusion criteria: (1) born in Italy and therefore possessing uniform vaccination cards, (2) able to present a recent vaccination certificate issued by the Public Health Office, and (3) have a quantitative assay of antibodies against rubella.

The study involved 3,759 males (34.1%) and 7,263 females (65.9%) enrolled in medical and surgical degree courses (4,922, 44.7%), dentistry (334, 3.0%), and health professions (5,766, 52.3%). Geographically, most students originated from Northern Italy (93.6%), particularly the Veneto region (85.6%). Data were collected during health surveillance in compliance with legislative decree 81/08 and European Community Directive 90/679.

### Antibody Measurement

Antirubella IgG antibody titer was measured using the EIA Enzygnost method (Dade Behring, Marburg, Germany), and the results are reported as positive (>10 IU/mL), negative (<4 IU/mL), or equivocal (4–10 IU/mL). Antibody levels were examined in relation to history of disease, vaccination, or both. Equivocal results were statistically processed as negative according to Centers for Disease Control and Prevention (CDC) recommendations (11).

### Statistics

The 2 × 2 chi-square (χ^2^) test (Yates correction) was used to compare the differences in the prevalence of positive antibodies. Comparisons between means were made using the unpaired *t*-test (assuming unequal variances). The linear regression coefficient *r* (Pearson's product–moment correlation coefficient) was calculated to correlate single independent variables with rubella antibody titer. Multiple linear regression analysis was employed to identify the variables affecting antibody level (dependent variable), such as (independent variables) sex, year of birth, and the number of vaccine doses received (none, one, or two). In all regression analyses (linear and multiple), the antibody titer data were log_10_-transformed due to the asymmetric distribution. Furthermore, 7 year-of-birth groups were established: before 1960, between 1961 and 1969, 1970 and 1979, 1980 and 1985, 1986 and 1990, 1991 and 1995, and after 1995. Other statistical analyses were descriptive. Significance is stated as *p* < 0.05. Statsdirect version 2.7.7 (Statsdirect Ltd., Birkenhead, Merseyside, UK) was used for statistical analyses.

## Results

**Figure 1:**
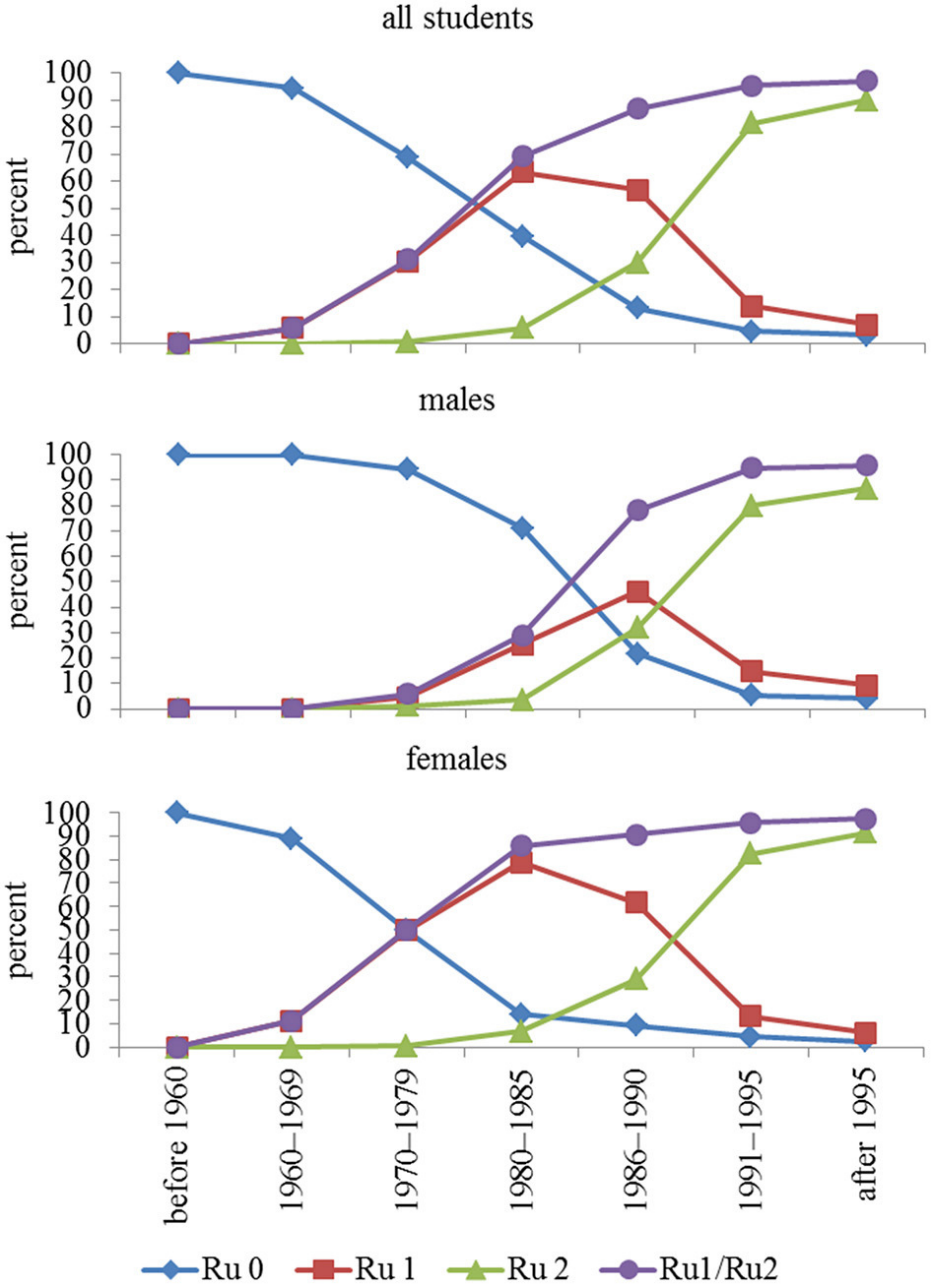
Compliance with rubella vaccination in relation to year-of-birth group. In addition to no vaccination, one dose, and two doses, a fourth option is receiving the vaccine (regardless of whether one or two doses). Ru = rubella, Ru 0 = unvaccinated, Ru 1 = one dose, Ru 2 = two doses, Ru1/Ru2 = vaccinated once and twice considered together.

Compliance with rubella vaccination began to increase in subjects born in the decade 1970–1979, almost exclusively for women, who reached ~50% vaccination coverage (one dose). In subsequent years, a progressive increase in vaccination compliance was observed in males, reaching a coverage (at least one dose) close to 100% (97%), as shown in Figure 1. Overall, 9,618 students were vaccinated (87.3%), of which 3,236 (29.4%) had one dose and 6,382 (57.9%) had two doses.

The vaccine, even after one dose, exhibited not only high coverage (97%), but also high seropositivity (>90%), even if the antibody titer, after both one and two doses, progressively declined in younger subjects.

Table 1 shows the differences in seropositivity and antibody titer between males and females; when considered collectively and subdivided according to the vaccination schedules, females were significantly more responsive than males with both one and two vaccine doses. It is also of interest that between those vaccinated, there was no statistically significant difference in the percentage of positives between those receiving one or two doses, but paradoxically, antibody titer was higher (1.67 times) in those vaccinated with one dose, even though the time between last vaccine dose and analysis was almost 4 years less in those vaccinated twice than in those vaccinated once.

**Table 1:** Seropositivity and antibody titer in unvaccinated students and students vaccinated with one or two doses.

					**Titer IU/mL**		Time^*^
**No vaccine**	***N***	**Positives**	**%**	***p***	**Mean ± SD**	***p***	**Years**
All	1,404	1,218	86.8		129.8 ± 106.1		
Males	792	698	88.1		136.7 ± 109.8		
Females	612	520	85.0	n.s.	120.8 ± 100.5	0.0048	
**One dose**							
All	3,236	3,157	97.6		105.4 ± 83.9		13.8 ± 5.1
Males	776	735	94.7		82.1 ± 80.0		15.7 ± 5.3
Females	2,460	2,422	98.5	<0.0001	112.7 ± 83.8	<0.0001	13.2 ± 4.9
**Two doses**							
All	6,382	6,192	97.0		63.2 ± 58.0		10.1 ± 3.0
Males	2,191	2,101	95.9		58.2 ± 56.6		10.3 ± 2.9
Females	4,191	4,091	97.6	0.0002	65.8 ± 58.6	<0.0001	10.1 ± 3.0

*Statistical significance refers to the comparison between males and females*.

* *Time was determined based on the date of vaccination if vaccinated once and on the second dose if vaccinated twice*.

However, by categorizing the students by year of birth and sex, even if a greater response from females was observed, it appeared less consistent and only in some year-of-birth groups (Table 2).

**Table 2:** Characteristics of antibodies against rubella in terms of positivity percentage and antibody titer.

**Year of birth**						**Titer IU/mL**	
**No vaccine**		***N***	**Positives**	**%**	***p***	**Mean ± SD**	***p***
Before 1960	All	14	14	100.0		101.5 ± 102.6	
	Males	7	7	100.0		104.7 ± 120.4	
	Females	7	7	100.0	n.s.	98.3 ± 91.1	n.s.
1960–1969	All	66	66	100.0		109.2 ± 69.8	
	Males	34	4	100.0		108.5 ± 69.1	
	Females	32	32	100.0	n.s.	109.9 ± 71.7	n.s.
1970–1979	All	276	274	99.3		130.2 ± 84.9	
	Males	162	160	98.8		141.6 ± 91.4	
	Females	114	114	100.0	n.s.	114.0 ± 71.9	n.s.
1980–1985	All	451	444	98.4		146.6 ± 96.2	
	Males	304	297	97.7		151.0 ± 99.8	
	Females	147	147	100.0	n.s.	137.4 ± 88.0	n.s.
1986–1990	All	336	317	94.3		162.9 ± 114.3	
	Males	173	162	93.6		162.8 ± 123.9	
	Females	163	155	95.1	n.s.	162.9 ± 103.5	n.s.
1991–1995	All	182	86	43.3		81.4 ± 119.6	
	Males	75	33	44.0		84.7 ± 128.3	
	Females	107	53	49.5	n.s.	79.1 ± 113.8	n.s.
after 1995	All	79	17	21.5		25.9 ± 73.9	
	Males	37	5	13.5		13.9 ± 52.4	
	Females	42	12	28.6	n.s.	36.4 ± 88.0	n.s.
**One dose**							
before 1960^*^	All	0					
1960–1969	All	4	4	100.0		90.4 ± 67.5	
	Males	0					
	Females	4	4	100.0		90.4 ± 67.5	
1970–1979	All	122	122	100.0		144.0 ± 85.3	
	Males	8	8	100.0		118.2 ± 83.9	
	Females	114	114	100.0	n.s.	145.9 ± 85.5	n.s.
1980–1985	All	929	925	99.6		121.6 ± 77.9	
	Males	108	107	99.1		105.0 ± 76.3	
	Females	821	818	99.6	n.s.	123.8 ± 77.9	n.s.
1986–1990	All	1453	1419	97.7		107.9 ± 88.1	
	Males	366	346	94.5		89.0 ± 84.1	
	Females	1087	1073	98.7	<0.0001	114.3 ± 88.6	<0.0001
1991–1995	All	542	516	95.2		80.5 ± 76.9	
	Males	210	196	93.3		72.3 ± 79.9	
	Females	332	320	96.4	n.s.	85.6 ± 74.5	0.0496
after 1995	All	186	171	91.9		52.1 ± 50.9	
	Males	84	78	92.9		44.0 ± 41.4	
	Females	102	93	91.2	n.s.	58.8 ± 56.8	0.0421
**Two doses**							
Before 1960^*^	All	0					
1960–1969^**^	All	0					
1970–1979	All	3	3	100.0		107.0 ± 78.5	
	Males	2	2	100.0		112.0 ± 110.3	
	Females	1	1	100.0		97	
1980–1985	All	86	86	100.0		86.3 ± 51.3	
	Males	15	15	100.0		86.5 ± 50.4	
	Females	71	71	100.0	n.s.	86.3 ± 51.9	n.s.
1986–1990	All	765	754	98.6		71.9 ± 61.3	
	Males	252	249	98.8		69.3 ± 59.3	
	Females	513	505	98.4	n.s.	73.2 ± 62.2	n.s.
1991–1995	All	3,182	3,136	98.6		69.4 ± 61.5	
	Males	1,130	1,101	97.4		63.3 ± 59.3	
	Females	2,052	2,035	99.2	0.0002	72.8 ± 62.5	<0.0001
After 1995	All	2,346	2,213	94.3		51.0 ± 49.5	
	Males	792	734	92.7		46.7 ± 49.5	
	females	1,554	1,479	95.2	0.0174	53.1 ± 49.4	0.0031

*The data are divided by groups of date of birth, sex, and number of doses of vaccine*.

* *No student born before 1960 has had the rubella vaccine*;

** *no student born before 1969 has had two doses of the vaccine. Statistical significance refers to the comparison between males and females*.

**Figure 2:**
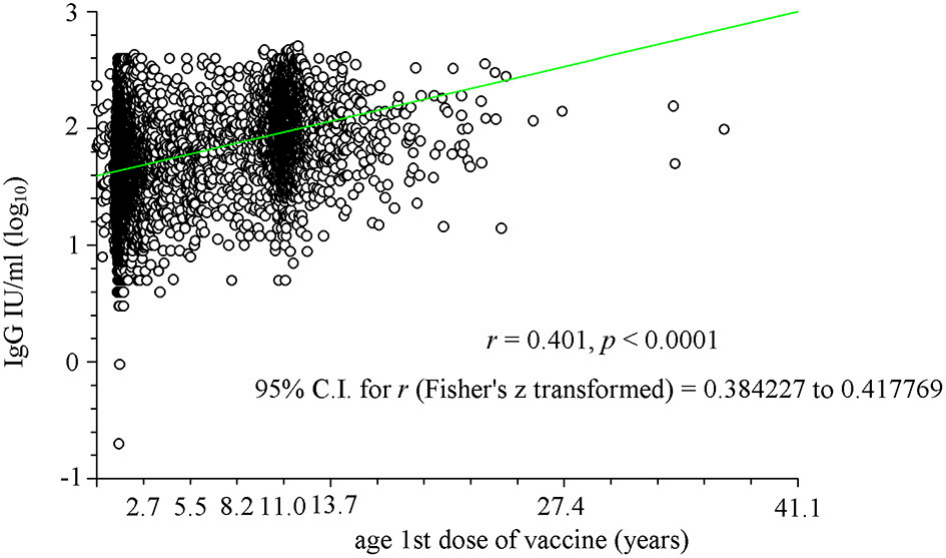
Linear regression analysis of the relationship between age at which the first vaccine dose was administered (in years) and logarithm of antibody titer (IgG). Two particular age groups have been identified: between 1 and 2 years and that of ~11 years. The first age group consisted of subjects who afterwards received a second dose of vaccine, whereas the second brings together both those who received only one dose during adolescence (particularly females) and those who received the second dose between the age of 8 and 11 years.

To better highlight this relationship, the two parameters (antibody titer and age at first dose) were plotted (Figure 2). A significant correlation was observed (*r* = 0.389, *p* < 0.0001) with two particular age groups: between 1 and 2 years and that of ~11 years. The first probably consisted of subjects who afterward received a second dose of vaccine, whereas the second brings together both those who received only one dose during adolescence (particularly females) and those who received the second dose between the age of 8 and 11 years. The significant effect of the time between receiving the vaccine and the date of the analysis (data not shown) was significant (*p* < 0.0001) only from a statistical point of view due to the large number of samples, but it was not significant from an objective point of view (*r* = −0.071).

**Figure 3:**
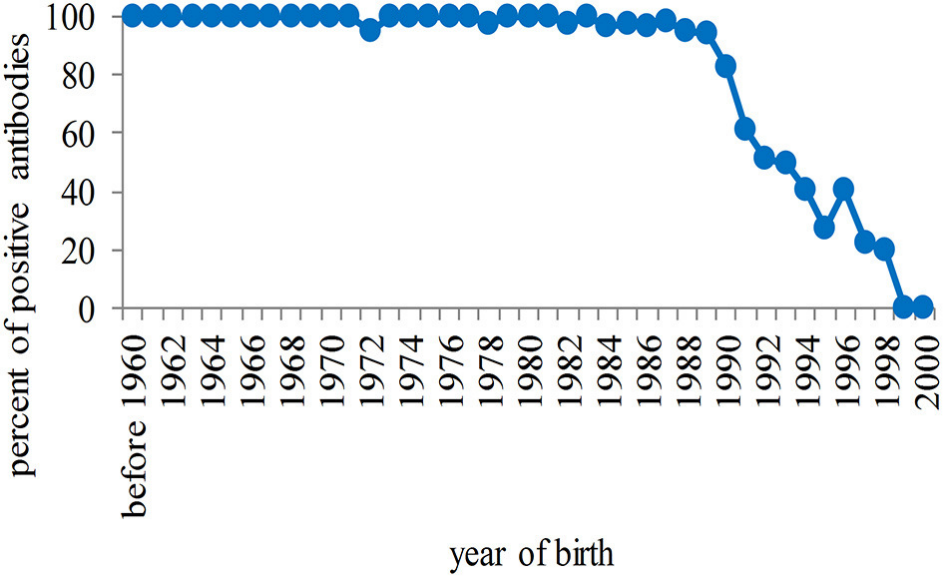
Percentage of seropositivity of antibodies against rubella in unvaccinated students in relation to the year of birth. The rate of positivity is high in students born before 1990 and progressively tends to zero in the population born after that year.

Among unvaccinated students, a high rate of positivity for antibodies against rubella was observed in those born before 1990, although the number was dramatically reduced among females (due primarily to high compliance), and then seropositivity progressively declined to zero among younger students (Figure 3).

Multivariate analyses highlighted the effect of independent variables on antibody titer developed both after infection and after vaccination (Table 3). In panel A, all students were evaluated, including unvaccinated students: Year of birth, sex, and vaccination showed a significant effect on the antibody titer. In panel B, where the effect of independent variables was evaluated only in vaccinated students, the age at which the first dose of vaccine was administered is of particular interest. The number of vaccine doses administered did not affect the antibody titer.

**Table 3:** Panel A: Multiple linear regression analysis for all students, unvaccinated and vaccinated (once or twice); Panel B: The analysis was performed only for vaccinated subjects (once or twice).

**Panel A**	**b**	***r***	**t**	***p***
Intercept	54.471946		34.669043	<0.0001
Year of birth	**−0.026523**	**−0.303853**	**−33.477332**	**<0.0001**
Sex	**0.075356**	**0.09051**	**9.533432**	**<0.0001**
Vaccination	**−0.020842**	**0.028648**	**3.008307**	**0.0026**
**Panel B**				
Intercept	2.099115		50.369121	<0.0001
Year of birth	**−0.074095**	**−0.160494**	**−15.941666**	**<0.0001**
Sex	**0.0666**	**0.089416**	**8.801643**	**<0.0001**
Vaccination	**–**0.015588	**–**0.011065	**–**1.084844	0.278
Age 1st dose	**0.000057**	**0.15002**	**14.876456**	**<0.0001**
Time	**−0.000028**	**−0.091667**	**−9.025113**	**<0.0001**

*Time is the interval between the last dose of vaccine and the measurement of antibodies (logarithmic transformation). Significant results are in bold*.

Finally, a comparative analysis of seropositivity and antibody titer after the administration of the vaccine alone or in the MMR formulation was performed (Table 4). The effect of the vaccine alone (one dose) or the vaccine alone plus MMR (two doses) exhibited a more significant response in terms of seropositivity and antibody titer (*p* < 0.0001). It should be noted that the vaccine alone was used almost exclusively in females and before the year 2000. Only one student was vaccinated with two doses of rubella vaccine alone.

**Table 4:** Comparative analysis of seropositivity and antibody titer after administration of the vaccine alone or in the MMR formulation.

					**Titer IU/mL**		Time^*^
**One dose**	***N***	**Positives**	**%**	***p***	**mean ± SD**	***p***	**Years**
Rubella alone	1,049	1,046	99.7		129.4 ± 81.3		12.7 ± 4.5
MMR	2,187	2,111	93.9	<0.0001	93.9 ± 82.7	<0.0001	14.3 ± 5.2
**Two doses**							
Rubella alone +MMR	110	110	100.0		101.8 ± 72.4		8.4 ± 3.8
MMR+MMR	6,272	6,082	97.0	n.s.	62.5 ± 57.5	<0.0001	10.2 ± 2.9

*Statistical significance refers to the comparison between rubella vaccine alone (or alone plus MMR) and MMR formulation*.

* *Time was determined based on the vaccination date if vaccinated once and on the second dose if vaccinated twice*.

## Discussion

Rubella vaccination coverage appears to be optimal, especially for those born after 1985 (with at least one dose, >90%), reaching 97.5% in those born after 1995. Of interest is the fact that in those born before 1990, there is a significant discrepancy in vaccination compliance between males and females, with females much more compliant. This is likely related to two main factors: (1) the awareness that women of child-bearing age are at higher risk of CRS in the event of infection and (2) the active on-site supply of rubella vaccinations for teenage females during the last year of primary and first year of secondary school from 1972 to the 1988–1989 cohort.

Of further interest is evidence that seropositivity was high (>95%) in those born before 1990, even if not vaccinated. For those born in subsequent years, seropositivity rapidly declined to <20% and then to zero. This means a significant decrease in circulation of the wild virus and therefore a lack of natural boosters, which is probably the cause of the progressive reduction in antibody titer after vaccination in the youngest. On the other hand, the immune response to a single dose of vaccine is optimal, such that it alone achieves and exceeds herd immunity, which for rubella has been calculated at between 85 and 87% (12). It is therefore evident that between natural immunity and immunity acquired with the vaccine, the population of future HCWs will have an immunization rate close to 100%. On the other hand, recent research in a cohort of female HCWs demonstrated that ~10% had non-protective antibodies, suggesting a third dose of the vaccine would be needed in these cases (13).

The high seropositivity in a very large cohort confirms that the rubella vaccine is particularly effective (14–18); the efficacy of one dose is >95%, and if high coverage is achieved, only one dose is required to achieve rubella elimination (19, 20). Indeed, two doses of the vaccine do not increase seropositivity and surprisingly result in a lower antibody titer. An adequate explanation is not at this moment available, except considering two factors: (1) One dose was administered at an older age (around 8 years) than the first dose, when the vaccine was administered twice (~2 years of age), and (2) the greater efficacy of the vaccine alone, widely used in the past, especially in females, in the single-dose vaccination schedule compared to the combined MMR vaccine, as already demonstrated for that vaccine against measles (21). Furthermore, our results clearly demonstrate that the antibody titer is significantly greater when the vaccine is administered in adolescence compared with that in childhood. Because infants lose maternally acquired immunity within 9 months of birth (22), vaccination is important to prevent rubella above all in women of child-bearing age (23).

Our results show that sex influences the antibody response, significantly higher in females, in terms of both seropositivity and antibody titer, with one or two doses. However, this difference is less consistent in relation to the years of birth, and males have a longer time interval between vaccine and serological analysis, especially if vaccinated with a single dose. The adaptive immunity in females is greater (24), and better vaccination response has been demonstrated for some vaccine types (25–27), but is not consistent for rubella (28) or for chickenpox (29).

The present research has the following weakness: Only the antibody titer was determined and not neutralizing antibodies. However, in such a large cohort, that analysis was economically impractical.

## Conclusions

In conclusion, the results of this study show that both the vaccination and the immune coverage of future HCWs against rubella are optimal, well above that required for herd immunity. The coverage also includes the male sex, which in the past was not considered necessary, as rubella is a generally benign disease, with the understanding that the eradication of rubella, and therefore of congenital rubella, did not pass only from females and that males play a role, if not vaccinated, in keeping the wild virus in circulation. Based on data provided by the Istituto Superiore di Sanità, Italy is approaching the eradication of congenital rubella, and the complete immunization of HCWs is a good start. The modest difference in the sex-related immune response to the vaccine does not appear substantial from our point of view.

## Data Availability Statement

The raw data supporting the conclusions of this article will be made available by the authors, without undue reservation.

## Ethics Statement

Ethical review and approval was not required for the study on human participants in accordance with the local legislation and institutional requirements. The patients/participants provided their written informed consent to participate in this study.

## Institutional Review Board Statement

This was an observational study in which we analyzed data obtained from a mandatory health surveillance of workers exposed to biological risks regulated by Italian legislative decree 81/2008; consequently, evaluation by an ethics committee was not necessary.

## Author Contributions

AT, CB, and PM involved in conceptualization, writing the review, and editing. CB involved in methodology. AT and PM involved in validation. AT involved in formal analysis, data curation, and supervision. AT, AN, and SM involved in investigation. AT and CB involved in writing the original draft preparation. All authors have read and agreed to the published version of the manuscript.

## Conflict of Interest

The authors declare that the research was conducted in the absence of any commercial or financial relationships that could be construed as a potential conflict of interest.

## Publisher's Note

All claims expressed in this article are solely those of the authors and do not necessarily represent those of their affiliated organizations, or those of the publisher, the editors and the reviewers. Any product that may be evaluated in this article, or claim that may be made by its manufacturer, is not guaranteed or endorsed by the publisher.
